# Food Access, Availability, and Affordability in 3 Los Angeles Communities, Project CAFE, 2004-2006

**Published:** 2010-02-15

**Authors:** Mark Vallianatos, Andrea Misako Azuma, Susan Gilliland, Robert Gottlieb

**Affiliations:** Urban and Environmental Policy Institute, Occidental College; Kaiser Permanente Southern California, Pasadena, California. At the time of the study, Ms Azuma was affiliated with Occidental College, Los Angeles, California; University of Southern California, Los Angeles, California; Occidental College, Los Angeles, California

## Abstract

**Introduction:**

Racial/ethnic minority communities are at increasingly high risk for chronic diseases related to obesity. Access to stores that sell affordable, nutritious food is a prerequisite for adopting a healthful diet. The objective of this study was to evaluate food access, availability, and affordability in 3 nonoverlapping but similar low-income communities in urban Los Angeles, California.

**Methods:**

Using a community-based participatory research approach, we trained community members to conduct a food assessment to 1) map the number and type of retail food outlets in a defined area and 2) survey a sample of stores to determine whether they sold selected healthful foods and how much those foods cost. We used descriptive statistics to summarize findings.

**Results:**

Of the 1,273 food establishments mapped in the 3 neighborhoods, 1,023 met the criteria of "retail food outlet." The most common types of retail food outlets were fast-food restaurants (30%) and convenience/liquor/corner stores (22%). Supermarkets made up less than 2% of the total. Convenience/liquor/corner stores offered fewer than half of the selected healthful foods and sold healthful foods at higher prices than did supermarkets.

**Conclusions:**

Access to stores that sell affordable healthful food is a problem in urban Los Angeles communities. Healthful food strategies should focus on changing food environments to improve overall community health.

## Introduction

In Los Angeles County, California, more than half of adults are overweight or obese (1). Obesity is more common among African Americans and Latinos than whites and Asians and is associated with low-income populations (2). Low-income racial/ethnic minority communities in Los Angeles County are at increasingly high risk for obesity-related chronic diseases such as type 2 diabetes and cardiovascular disease (3).

Diets that include fresh fruits, vegetables, and whole grains can reduce the risk for obesity and its consequences (4). People make food choices based not only on personal preference but on environmental factors such as food access, availability, and affordability. Food access refers to people's ability to reach local food retail outlets by using convenient modes of transportation. Some research has drawn distinctions between "potential access" where consumers could shop and "realized access" where consumers actually shop (5). Food availability refers to what healthful foods and beverages are sold or served at retail food outlets. Food affordability refers to the idea that low-income people must choose foods based on their price, not just relative to other foods but relative to competing necessities, such as housing, clothing, and transportation (6). Food justice is the concept that everyone deserves healthful food and that the benefits and risks associated with food should be shared fairly (7).

A healthful food environment is necessary for people to make healthier food decisions. An association between the food environment and meeting dietary recommendations has been documented; specifically, more fruits and vegetables were eaten in areas that had more supermarkets (8). A follow-up study found an association between supermarket concentration and lower prevalence of overweight and obesity, and between concentration of corner stores and a higher prevalence of overweight and obesity (9). Supermarket concentration also has been associated with lower body mass index and weight, and convenience store concentration with higher body mass index and weight (10).

Several studies have documented a relationship between income or ethnicity of neighborhood residents and concentration of retail food outlets. Low-income neighborhoods have fewer chain supermarkets than do middle-income neighborhoods, and African American and Hispanic neighborhoods have fewer chain supermarkets than do non-Hispanic white neighborhoods (11). Another study of African American populations found fewer healthful food options at restaurants in less affluent areas of Los Angeles (12).

Better availability of healthful foods, such as low-fat and high-fiber foods, has been associated with eating a more healthful diet (13), but availability varies by store type (14). Supermarkets are often able to sell a larger variety of foods at lower prices. Equivalent food items sold at smaller food stores can cost up to 75% more than at supermarkets (15), and quality can be lower (16).

Evaluating food access, availability, and affordability in a community is the first step toward improving these environmental factors. Community Action on Food Environments (Project CAFE) is a university-community partnership formed to improve food justice in specific neighborhoods (17). Project CAFE is managed by the Urban and Environmental Policy Institute (UEPI), a community-oriented research and advocacy organization at Occidental College in Los Angeles.

We used a community-based participatory research approach to investigate access to and availability and affordability of healthful foods in 3 nonoverlapping but similar low-income, primarily Latino communities in urban Los Angeles.

## Methods

Project CAFE was a partnership of UEPI, the University of Southern California's Department of Preventive Medicine, and 3 community organizations: Esperanza Community Housing Corporation, the Healthy School Food Coalition, and Blazers Youth Services Community Club. The community organizations helped design and implement the project. Project CAFE partners used a food assessment in 3 Los Angeles neighborhoods. A food assessment systematically examines a broad range of community food issues and assets to learn what actions are needed to make the community more food secure. SAS version 9.1 (SAS Institute, Cary, North Carolina) was used to analyze the data and generate descriptive statistics.

The study area was 3 communities in the south and central parts of Los Angeles. The boundaries of each area were defined by the community partners (Healthy School Food Coalition: the MacArthur Park/Pico Union area; Esperanza Community Housing Corporation: area near the University of Southern California; Blazers Youth Services Community Club, Inc: south Los Angeles). The 3 areas have similar economic and demographic profiles: high levels of poverty with predominantly Latino populations. For example, according to the 2000 US Census, 61% of residents of the Esperanza Community Housing Corporation area were born outside the United States, and 85% of residents older than 5 years spoke a language other than English at home. Approximately 82% were Latino, 7% white, 4% African American, 4% Asian, and 1% American Indian. Nearly one-third of families in this area had an annual household income below the poverty threshold, more than triple the national average of 9% (18).

The community food assessment consisted of 1) food mapping to document the number and types of all retail food outlets (ie, including restaurants) and 2) store surveys to assess availability, price, and quality of foods from a convenience sample of the 3 types of food stores (convenience/liquor/corner, specialty food, and supermarket). Quality was assessed for all food items by inspecting them (eg, for brown spots on apples or brown color on meat) and checking their expiration dates. The tools used for store surveys were adapted from the food assessment tools published by the US Department of Agriculture (USDA) Community Food Security Assessment Toolkit (19) and the Community Food Security Coalition.

Community volunteers were trained to conduct food assessments from 2004 to 2006. Mapping training covered how to collect data that included the name, type, address, and public health grade (where applicable) for all restaurants and stores selling food. Survey training covered specifics on product size and type and measures of quality. Approximately 50 volunteers were trained in food assessment and 40 actively participated. All training was finished before food mapping and store surveying began. Teams of 2 to 4 surveyors were deployed to the neighborhood streets and food stores.

We conducted a census of all places that sell food, including supermarkets, fast-food and full-service restaurants, bars, convenience/liquor/corner stores, carryout restaurants, and mobile food vendors. All retail food outlets were mapped by project staff and trained community members during the spring and summer of 2005. Community members identified the boundaries of their neighborhoods and created maps. Project staff and community members used the maps to walk the areas and locate the food establishments. They categorized retail food outlets according to the North American Industry Classification System codes and other methods (20), described each category, and documented the number of stores (Table 1).

The USDA's Thrifty Food Plan (21), which recommends healthful low-cost foods, was used to craft the survey tool to assess the availability and affordability of healthful foods in stores in the study areas. To reflect the demographics of the mapped communities, community participants and project staff chose appropriate Thrifty Food Plan items and added more ethnic and specialty foods to the Project CAFE survey (Appendix A). Community participants and project staff assessed food in 3 store types: convenience/liquor/corner, specialty food stores (ie, those specializing in one item, such as fish), and supermarkets. Price per given amount and quality of selected foods were documented.

A convenience sample of 10 community members and project staff were interviewed in 2 groups about their experiences of food shopping in the communities.

## Results

A total of 1,273 retail food outlets were mapped for the 3 areas. Of these, 1,023 fell into the following categories: supermarket, convenience/liquor/corner, convenience with gas, specialty food, full-service restaurant, fast-food restaurant, carryout, carryout specialty food, bar/tavern, or mobile food truck. The most common type of retail food outlet was fast-food restaurants, including carryout stores (30%) (Table 1). Of the 3 types of nonrestaurant food stores, convenience/liquor/corner stores (22%) were the most common, followed by specialty food stores (14%) and supermarkets (<2%). The 15 supermarkets available were in 2 of the communities; the third had no supermarkets. No farmers' markets were available. We found similarities in the distribution of the food establishments. In all 3 areas more than half of food establishments were convenience/liquor/corner stores or fast-food restaurants.

Food categories from the Thrifty Food Plan were modified to include more variety of ethnic food preferences and specialty items for our survey to assess the food stores. Sweets and calorie-dense snacks were included in the survey. The 3 communities surveyed a sample of their food stores (n = 90). Two communities surveyed 100% of their supermarkets; the third had none to survey. Community 1 surveyed 35% of its convenience/liquor/corner stores and 35% of its specialty stores; community 2 surveyed 88% of its convenience/liquor/corner stores and 100% of its specialty stores; and community 3 surveyed 10% of its convenience/liquor/corner stores and 5% of its specialty stores.

Availability, price, and quality varied by store type. Overall, fewer than half of all the stores surveyed carried a given item, yet nearly 100% of items were available at the supermarkets (Appendix B). Foods and beverages high in fat and sugar were generally more available in convenience/liquor/corner stores than fruits and vegetables. For example, 85% of surveyed convenience/liquor/corner stores sold Flaming Hot Cheetos and 89% sold Pepsi, but only 32% sold carrots and only 17% sold broccoli. Supermarkets offered the lowest price on the greatest number of healthful foods, including oatmeal, whole wheat bread, carrots, apples, 2% milk, and beef (Table 2). Convenience/liquor/corner stores offered a limited number of low-priced foods (corn tortillas, potatoes, oranges, and eggs). Specialty food stores offered the fewest low-priced foods (lettuce and chicken legs). All of the store types carried some items that were past their expiration dates and items that were rated poor to fair quality. Supermarkets carried the fewest expired or poor-quality items, and specialty food stores carried the most expired or poor-quality items.

Participants in small-group interviews described the following barriers to accessing healthful foods:

- Food is perceived to be expensive. Participants reported that they have a limited food budget, and although they want the highest quality food for their families, they have to settle for the quality they can afford.
- The nearest supermarkets are typically more than 1 mile away. Participants reported that many residents do not have their own transportation and must walk, ride a bus, or take a grocery store shuttle, which requires that they spend a minimum amount ($40). Residents who own cars are challenged by the price of gasoline and the inconvenience of driving farther to a supermarket. Many residents resort to shopping daily and purchasing small amounts of food from convenience stores or other small stores near their homes.
- Shopping after dark is considered unsafe because of violent crime in the 3 communities.
- Fast food can be easily purchased in the neighborhoods and outside the school grounds (which are subject to several mandates that limit the purchase of snack foods and sodas) because many mobile food vendors sell prepared foods in these areas.

## Discussion

We found that access to stores that sell healthful food is a problem in urban Los Angeles. Our results support the findings of studies of similar neighborhoods throughout the United States (22).

A study on food landscapes calculated the "retail food environment index" in California counties and cities (23) and found that Los Angeles has more than 4 times as many fast food and convenience stores as supermarkets and produce vendors. A follow-up study incorporated health survey data and showed that prevalence of obesity and diabetes was associated with this index (24). A study conducted after race riots in Los Angeles in 1992 found that supermarket chains had largely abandoned the inner city. For a period, food security advocates called for reinvestment in urban areas and implementation of a coordinated food policy (25). However, 10 years later, this "grocery gap" persisted; each supermarket in the areas of the 1992 riots (which overlap with the Project CAFE survey neighborhoods) served 27,986 people, compared with 18,649 people in Los Angeles County (26).

Our results show that low-income communities in Los Angeles have few supermarkets and limited access to healthful food items including fresh, high-quality foods. Participants in group interviews perceived that the nearest supermarkets were more than 1 mile away from their homes, 2 to 4 times as far as planners typically consider to be within reasonable walking distance (27). The most common type of food store in the 3 neighborhoods was the convenience/liquor/corner store. Healthful food items were often unavailable at these stores, but high-sugar and calorie-dense snacks were readily available. Many community members rely on the most convenient retail food outlets, including convenience/liquor/corner stores, full-service restaurants, specialty food stores, and fast-food restaurants, as their primary food sources. The availability of healthful foods at these facilities is limited. The local food environment is therefore likely to limit residents' ability to make healthful dietary choices.

This study has limitations. It is a cross-sectional study that provides a snapshot of the communities at 1 point in time. Furthermore, it is limited to urban Los Angeles and findings may not be generalizable to other areas. Nevertheless, we demonstrate that residents of south and central Los Angeles may face barriers to purchasing healthy foods in terms of access, availability, and affordability. These barriers may contribute to the development of chronic diet-related diseases, such as obesity, diabetes, and cardiovascular disease.

Because of the lack of supermarkets in low-income communities, residents sometimes must shop for food at smaller stores where prices are higher and quality lower. Residents of these communities should be educated to make better lifestyle choices, but access barriers such as limited food retail outlets cannot be ignored. More awareness of the barriers, continued community engagement, support from elected officials and economic development agencies, and interventions to change policies, land-use patterns, and market trends are needed. Project CAFE has developed targeted campaigns to make the participating communities healthier by working to attract new supermarkets, working with corner stores to offer more fresh fruits and vegetables, starting farmers' markets on school sites, working to incorporate food access goals and policies into community plans or zoning regulations, and creating a Los Angeles network of community groups to work on food access policies. Policies adopted in other areas, such as the Pennsylvania Fresh Food Financing Initiative, designed to subsidize new food markets (28), and New York City's Green Carts program, creating special permits for mobile food vendors offering fresh produce in low-income areas (29), are models for potential interventions in Los Angeles.

## Figures and Tables

**Table 1:** Retail Food Outlets Mapped (N = 1,023), Project CAFE, Los Angeles, California, 2004-2006

**Category**	**Defining Characteristics**	**No. (%)^a^**
Fast-food restaurant/carryout /carryout specialty	Fast-food restaurant: part of a chain that sells fast food. Food is served on trays and ordered at a counter. Carryout: sells fast food at a counter that is taken away. Carryout specialty: carryout that specializes in coffee, doughnuts, smoothies, or ice cream.	303 (30)
Convenience/ liquor/corner store	May or may not be part of a chain. Smaller than a supermarket. Sells smaller variety than supermarkets.	223 (22)
Full-service restaurant	Can be local or part of a chain. Table service is available.	171 (17)
Specialty food store	Meat market (carnecería), fish market, bakery (panadería), or other kind of store specializing in a single item or type of item.	140 (14)
Mobile food truck	Sells food from wheeled vehicles, carts, and other mobile sites.	115 (11)
Convenience store with gas	Sells food and convenience items as well as gasoline.	39 (4)
Bar or tavern	Sells alcohol.	17 (2)
Supermarket	A chain store that sells a wide variety of general food items.	15 (2)

Abbreviation: CAFE, Community Action for Food Environments.

a Percentages do not total 100 because of rounding.

**Table 2:** Prices of Selected Foods^a^ in 90 Surveyed Food Stores, Project CAFE, Los Angeles, California, 2004-2006

Food Category	Overall Mean Price, $ (Range)	Supermarket Mean Price, $ (Range)	Convenience/Liquor/Corner Store Mean Price, $ (Range)	Specialty Food Store Mean Price, $ (Range)
**Grains**				
Oatmeal/18 oz	2.43 (0.98-5.12)	2.00 (0.98-3.00)	2.70 (1.39-5.12)	2.38 (1.10-4.56)
Whole-wheat bread/24 oz loaf	2.30 (0.99-3.35)	2.19 (0.99-3.29)	2.49 (1.79-3.35)	2.20 (1.30-2.89)
Corn tortillas/12 oz taco size	0.66 (0.29-2.59)	0.66 (0.29-1.59)	0.63 (0.29-2.59)	0.66 (0.33-1.16)
**Vegetables**				
Carrots/lb	0.49 (0.20-1.39)	0.37 (0.20-0.50)	0.52 (0.30-1.00)	0.62 (0.33-1.39)
Lettuce/head	0.75 (0.25-1.30)	0.84 (0.39-1.29)	0.70 (0.25-1.29)	0.68 (0.45-1.30)
Potatoes/5 lb	1.57 (0.37-3.95)	1.91 (0.75-3.95)	1.66 (0.37-3.45)	1.91 (0.37-3.45)
**Fruit**				
Apples/lb	0.63 (0.22-1.00)	0.57 (0.33-0.99)	0.69 (0.22-1.00)	0.59 (0.50-0.69)
Oranges/lb	0.47 (0.11-1.99)	0.59 (0.20-1.99)	0.38 (0.20-0.70)	0.41 (0.11-0.89)
Avocados/each	0.91 (0.33-1.75)	0.68 (0.33-1.25)	1.01 (0.50-1.75)	1.07 (0.50-1.50)
**Dairy**				
Low-fat (2%) milk/gallon	3.17 (2.25-4.50)	3.00 (2.25-3.49)	3.26 (2.25-4.50)	3.14 (2.49-3.95)
Cheddar cheese/lb	4.39 (1.60-9.16)	3.50 (1.60-5.69)	4.94 (2.25-9.16)	4.44 (3.59-5.13)
**Meat and meat alternatives**				
Chicken legs/lb	1.18 (0.39-2.39)	1.30 (0.39-2.39)	1.87 (0.79-1.99)	1.26 (0.79-1.99)
Beef/lb	2.38 (1.39-3.29)	2.34 (1.39-3.29)	2.43 (1.99-2.99)	NA^b^
Eggs/dozen	1.54 (0.40-2.99)	1.61 (0.79-2.59)	1.49 (0.40-2.29)	1.62 (1.00-2.99)
Pinto beans/lb	1.02 (0.44-3.49)	0.95 (0.38-1.69)	1.02 (0.44-3.49)	1.10 (0.69-3.00)

Abbreviations: CAFE, Community Action for Food Environments; NA, not assessed.

a Selected from the US Department of Agriculture Thrifty Food Plan (21) and community input on ethnic specialty foods.

b Fewer than 5 specialty food stores offered beef.

## Appendices

### Appendix A. Foods Assessed^a^ in 90 Surveyed Food Stores, Project CAFE, Los Angeles, California, 2004-2006

**Table:**

**Category**	**Food**
Grains	Breads, yeast and quick; breakfast cereals, cooked and ready-to-eat; rice and pasta; flours; grain-based snacks and cookies
Vegetables	Potatoes; dark green and deep yellow vegetables; other vegetables
Fruits	Citrus, melons, berries and juices; noncitrus fruits and juices
Dairy	Whole milk, yogurt, cream; low-fat and skim milk, low-fat yogurt; cheese; milk drinks and milk desserts
Meat and meat alternatives	Beef, pork, veal, lamb, and game; chicken, turkey, and game birds; fish and fish products; bacon, sausages, luncheon meats; eggs and egg mixtures; dried beans, lentils, peas, and nuts; tofu
Other foods	Table fats, oils, and salad dressings; gravies, sauces, condiments, spices, salt; coffee and tea; fruit drinks, soft drinks; sugars, sweets, and candies

Abbreviation: CAFE, Community Action for Food Environments.

a Based on US Department of Agriculture Thrifty Food Plan (21) and community input on ethnic specialty foods.

### Appendix B. Foods Unavailable^a^ in at Least Half of 90 Surveyed Food Stores, Project CAFE, Los Angeles, California, 2004-2006

**Table:**

**Category**	**Food**
Grains	Brown rice, whole-wheat bread, ready-to-eat cold cereals, grits
Vegetables	Broccoli, cabbage, carrots, cauliflower, cucumbers, green peppers, jicama, lettuce, potatoes
Fruits	Avocados, melons, apples, papayas, mangos, oranges, limes
Dairy and dairy alternatives	Soy milk, soy cheese, cheddar, string cheese
Meat and meat alternatives	Ground beef, turkey, lunch meat, chicken legs, chicken breast, tofu

Abbreviation: CAFE, Community Action for Food Environments.

a On day of survey, all stores combined.

## References

[1] Obesity on the rise. L.A. Health 2003.

[2] Hung HC, Joshipura KJ, Jiang R, Hu F, Hunter D, Smith-Warner S (2004). Fruit and vegetable intake and risk of major chronic disease. J Natl Cancer Inst.

[3] Diabetes on the rise in Los Angeles County adults. L.A. Health Trends 2007.

[4] Morland K, Wing S, Diez Roux (2002). The contextual effect of the local food environment on residents' diets: the Atherosclerosis Risk in Communities Study. Am J Public Health.

[5] Sharkey JR, Horel S Characteristics of potential spatial access to a variety of fruits and vegetables in a large rural area. Paper presented at: Understanding the Economic Concepts and Characteristics of Food Access.

[6] Ver Ploeg M, Breneman V, Farrigan T, Hamrick K, Hopkins D, Kaufman P (2009). Access to affordable and nutritious food — measuring and understanding food deserts and their consequences: report to Congress.

[7] Gottlieb R, Joshi A (2010). Food justice.

[8] Morland K, Diez Roux, Wing S (2006). Supermarkets, other food stores, and obesity: the Atherosclerosis Risk in Communities Study. Am J Prev Med.

[9] Powell LM, Auld C, Chaloupka FJ, O'Malley PM, Johnston LD (2007). Associations between access to food stores and adolescent body mass index. Am J Prev Med.

[10] Powell LM, Slater S, Mirtcheva D, Bao Y, Chaloupka FJ (2007). Food store availability and neighborhood characteristics in the United States. Prev Med.

[11] Cheadle A, Psaty BM, Curry S, Wagner E, Diehr P, Koepsell T (1991). Community-level comparisons between the grocery store environment and individual dietary practices. Prev Med.

[12] Lewis LB, Sloane D, Nascimento LM, Diamant A, Guinyard J, Yancey A (2005). African Americans' access to healthy food options in south Los Angeles restaurants. Am J Public Health.

[13] Mikkelsen L, Chehimi S (2007). The links between the neighborhood food environment and childhood nutrition.

[14] Connell CS, Yadrick K, Simpson P, Gossett J, McGee BB, Bogle ML (2007). Food supply adequacy in the Lower Mississippi Delta. J Nutr Educ Behav.

[15] Wilson LA (1994). Nutrition and food cost study.

[16] Weinberg Z (2000). No place to shop: food access lacking in the inner city. Race, Poverty and the Environment.

[17] Azuma A (2007). Food access in central and south Los Angeles: mapping injustice, agenda for action.

[18] Census 2000 demographic profile highlights. Zip code tabulation area 90007.

[19] Cohen B (2000). Community Food Security Assessment Toolkit. E-FAN 02-013 [serial online].

[20] Morland K, Wing S, Diez Roux, Poole S (2002). Neighborhood characteristics associated with location of food stores and food service places. Am J Prev Med.

[21] Carlson A, Lino M, Juan W, Hanson K, Basiotis P (2007). Thrifty food plan 2006.

[22] Cotterill RW, Franklin AW (1995). The urban grocery store gap.

[23] (2007). Searching for healthful food: the food landscape in California cities and counties.

[24] (2008). Designed for disease: the link between local food environments and obesity and diabetes.

[25] Ashman L, De La Vega J, Dohan M, Fisher A, Hippler R, Romain B (1993). Seeds of change.

[26] Shaffer A (2002). The persistence of LA's grocery gap: the need for a new food policy and approach to market development.

[27] Fairfax County (Virginia) Planning Commission Transit-Oriented Development Committee. Walking distance research.

[28] (2008). Pennsylvania fresh food financing initiative: providing healthful food choices to Pennsylvania's communities.

[29] NYC Green Carts.

